# Relationship between hyperchloremia in braindead donors and delayed graft function in the kidney allograft recipients

**DOI:** 10.1186/2197-425X-3-S1-A459

**Published:** 2015-10-01

**Authors:** E Kieslichová, E Pokorná, M Protuš, O Viklický, E Uchytilová

**Affiliations:** 1 IKEM, KARIP, Prague, Czech Republic; 2 IKEM, Prague, Czech Republic

## Introduction

In the brain-dead organ donors a number of hormonal changes occurs, reflecting anterior and posterior pituitary failure. There is an early development of diabetes insipidus in almost 80% of brain-dead organ donors, characterized by inappropriate diuresis, severe hypovolemia, hyperosmolality, and electrolyte abnormalities, such as hypernatremia and hyperchloremia. Hypernatremia and hyperchloremia is often aggravated by infusions of large volumes of sodium and chloride-containing solutions, as a part of the therapy of intracranial hypertension.

## Objectives

As previously described in both animal and human studies, hyperchloremia alters renal functions by causing renal vasoconstriction and decrease in glomelural filtration. The aim of this study was to determine the relationship between the level of serum chlorides of the donor and the onset of the function of the kidney allograft.

## Methods

We retrospectively studied 52 transplanted kidney allografts from cadaveric donors. The effect of serum levels of chlorides of the donors on the recipient's serum creatinine levels on the first day and one week after transplantation was assessed. The incidence of acute tubular necrosis and the need for hemodialysis during the first week after transplantation was also monitored.

## Results

The mean level of serum chlorides of the donors was 117.92 mmol/l (+/- 10.82 s.d.). 39 of them had hyperchloremia (Cl->110 mmol/l). The average serum creatinine level of the recipients with hyperchloremic kidney allografts one day after transplantation was 515.07 umol/l +/- 186.55 s.d., and 195.55 umol/l +/-94.08 s.d. after one week. Serum creatinine level of the recipients with normochloremic kidney allografts one day after transplantation was 532.84 umol/l +/- 221.37 s.d., and 312.52 umol/l +/-217.13 s.d. after one week. Neither creatinine levels of the recipients on the first day (r = 0.06), nor one week after transplantation (r=-0.1) correlated significantly with the levels of serum chlorides of the donors. The need for hemodialysis or the incidence of acute tubular necrosis was not related to the degree of hyperchloremia of the donor either.

## Conclusions

In this study, statistically significant correlation between serum chloride levels of the brain-dead donors and the onset of the function of kidney allografts in the recipients was not found.
